# Nursing Home Visitor Policy and COVID-19 Infection Rates: A Marginal Structural Model Analysis

**DOI:** 10.1093/geroni/igaf122.1943

**Published:** 2025-12-31

**Authors:** Huiwen Xu, John Bowblis, Shuang Li, Jennifer Heston-Mullins, Yong-Fang Kuo, James Goodwin

**Affiliations:** Emory University, Atlanta, Georgia, United States; Miami University, Oxford, Ohio, United States; UTMB Health, The University of Texas Medical Branch, Galveston, Texas, United States; Miami University, Oxford, Ohio, United States; University of Texas Medical Branch, Galveston, Galveston, Texas, United States; UTMB Health, The University of Texas Medical Branch, Galveston, Texas, United States

## Abstract

During the COVID-19 pandemic, Ohio was the only state that collected facility-level visitation data after rescinding its ban on visitors. This study examines the association of allowing outside visitors with COVID-19 infection rates among nursing home residents. We assembled a cohort of Ohio nursing homes over 9 weeks (11/1/2020 to 1/3/2020). For each week, we obtained whether a facility allowed visitors, any COVID-19 infections among residents, community infection rates, and other facility characteristics. Marginal structural models examined the association of allowing visitors with resident infections, weighted by the inverse of the probability of allowing visitors. Of the 677 nursing homes with visitation data, the number of nursing homes that allowed visitors during any week from 10/29/2020 to 1/3/2021 ranged from 226 to 327, and the number with bans in place was between 316 and 448. Marginal models substantially improved the balance in both time-varying and time-invariant covariates between facilities that allowed vs. banned visitors. In the marginal models, allowing visitors was not associated with the unadjusted rates or adjusted odds of new infection among residents (odds ratio=0.92, 95% confidence interval: 0.78, 1.08). The result was similar in sensitivity analyses on the lagged effect of allowing visitors (odds ratio=1.02, 95% confidence interval: 0.86, 1.22). Allowing visitors in the context of adequate PPE and social distancing was safe, even in a period with large increases in community infection rates.

